# Comparative Study of Anterior Eye Segment Measurements with Spectral Swept-Source and Time-Domain Optical Coherence Tomography in Eyes with Corneal Dystrophies

**DOI:** 10.1155/2015/805367

**Published:** 2015-09-17

**Authors:** Anna K. Nowinska, Sławomir J. Teper, Dominika A. Janiszewska, Anita Lyssek-Boron, Dariusz Dobrowolski, Robert Koprowski, Edward Wylegala

**Affiliations:** ^1^Ophthalmology Clinic, Medical University of Silesia, 40-760 Katowice, Poland; ^2^Department of Ophthalmology, Saint Barbara Hospital, 41-200 Sosnowiec, Poland; ^3^Department of Biomedical Computer Systems, University of Silesia, Sosnowiec, Poland

## Abstract

*Purpose.* To compare anterior eye segment measurements and morphology obtained with two optical coherence tomography systems (TD OCT, SS OCT) in eyes with corneal dystrophies (CDs). *Methods*. Fifty healthy volunteers (50 eyes) and 54 patients (96 eyes) diagnosed with CD (epithelial basement membrane dystrophy, EBMD = 12 eyes; Thiel-Behnke CD = 6 eyes; lattice CD TGFBI type = 15 eyes; granular CD type 1 = 7 eyes, granular CD type 2 = 2 eyes; macular CD = 23 eyes; and Fuchs endothelial CD = 31 eyes) were recruited for the study. Automated and manual central corneal thickness (aCCT, mCCT), anterior chamber depth (ACD), and nasal and temporal trabecular iris angle (nTIA, tTIA) were measured and compared with Bland-Altman plots. *Results.* Good agreement between the TD and SS OCT measurements was demonstrated for mCCT and aCCT in normal individuals and for mCCT in the CDs group. The ACD, nTIA, and tTIA measurements differed significantly in both groups. TBCD, LCD, and FECD caused increased CCT. MCD caused significant corneal thinning. FECD affected all analyzed parameters. *Conclusions.* Better agreement between SS OCT and TD OCT measurements was demonstrated in normal individuals compared to the CDs group. OCT provides comprehensive corneal deposits analysis and demonstrates the association of CD with CCT, ACD, and TIA measurements.

## 1. Introduction

Corneal dystrophy (CD) is a group of inherited, bilateral, symmetric, slowly progressive corneal diseases without any relationship to environmental or systemic factors.

Noninvasive evaluation of anterior eye segment measurements is pertinent for the diagnosis of several corneal dystrophies types as well as other ophthalmic diseases, including glaucoma, keratoconus, and corneal degenerations, and is essential in planning corneal surgical and refractive procedures.

Optical coherence tomography (OCT), first introduced in 1991, is a high-speed, high-resolution, noncontact imaging technique developed for noninvasive cross-sectional imaging in biological systems [1]. The OCT technology has evolved from time-domain (TD OCT) to spectral-domain (SD OCT) and swept-source OCT (SS OCT). Anterior eye segment imaging with the 830 nm light wavelength TD OCT was demonstrated in 1994 [2]. Changing the light wavelength from 830 nm to 1310 nm allowed transscleral imaging with the scleral spur assessment [3]. TD OCT technology has a longitudinal resolution of 18 *μ*m and a transverse resolution of 60 *μ*m. It provides scans at a rate of up to 2048 A scans per sec. SD OCT, introduced in 2002, has an axial resolution of 5.0 *μ*m and a transverse resolution of 15 *μ*m [4, 5]. It scans at 26,000 A scans per sec and provides an increased signal-to-noise ratio and increased robustness compared with TD OCT [6]. SS OCT uses a monochromatic, tunable, fast-scanning laser source and a photodetector to detect wavelength-resolved interference signals [7, 8]. Commercially available SS OCT was introduced in 2008. It uses a swept-source laser wavelength of 1310 nm, scans up to 30,000 A scans per sec, and has longitudinal and transverse resolutions of 10 *μ*m and 30 *μ*m, respectively. The advantage of SS OCT is the simultaneous acquisition of numerous scans, which provides the possibility of creating a 3-dimensional corneal, anterior eye segment, or gonioscopy views. That feature could be especially important in eyes with corneal opacities to gain the possibility of creating a 3D pattern of the corneal changes.

OCT has been proven to provide reliable measurements of anterior eye segment parameters characterized by good repeatability and reproducibility [9–13]. Most SS OCT morphometry studies are based on normal subjects, with the exception of anterior chamber angle parameters in glaucomatous eyes [14–16] and corneal thickness measurements in keratoconic eyes [17, 18]. Currently, there are no data on anterior eye segment measurements with SS OCT in various corneal dystrophies. Previous papers on OCT imaging focused on describing corneal morphology features in different CDs [19–23]. The SS OCT was proved useful in planning of the phototherapeutic keratectomy to treat granular corneal dystrophy by determining the size, depth, and location of deposits based on the case report study [24]. The authors present a comprehensive, observational, comparative study of corneal thickness, anterior chamber depth, and trabecular iris angle measurements with TD OCT and SS OCT in eyes with corneal dystrophies compared to normal controls. Agreement between the TD OCT and SS OCT measurements is assessed.

## 2. Material and Methods

The study was conducted in accordance with the ethical standards stated in the 1964 Declaration of Helsinki and was approved by the Ethics Committee of the Medical University of Silesia, Katowice, Poland (KNE/0022/KB1/43/I/14). All patients had to sign informed consent before any study procedure.

Fifty healthy volunteers (50 eyes) and 54 patients (96 eyes) diagnosed with various corneal dystrophies (epithelial basement membrane dystrophy, EBMD = 12 eyes; Thiel-Behnke corneal dystrophy, TBCD = 6 eyes; lattice corneal dystrophy TGFBI type, LCD1 = 15 eyes; granular corneal dystrophy type 1, GCD1 = 7 eyes; granular corneal dystrophy type 2, GCD2 = 2 eyes; macular corneal dystrophy, MCD = 23 eyes; Fuchs endothelial corneal dystrophy, FECD = 31 eyes) were recruited for the study.

The inclusion criteria for the healthy subjects group were as follows: best corrected visual acuity of 20/20, refractive error less than or equal to ±3.0 D, and no history of ocular disease or surgery. The mean age of the subjects was 30 ± 7 years; there were 30 women and 20 men. The inclusion criteria for the study group included the clinical diagnosis of corneal dystrophy and no history of ocular surgery. The exclusion criterion was the presence of other ophthalmic or systemic diseases affecting corneal morphology. The mean age of the patients was 49 ± 16 years; there were 39 women and 15 men. 12 eyes of 12 patients with diagnosis of CD underwent keratoplasty procedures; therefore the eyes were excluded from the study group. The healthy subjects and the study group patients were age matched for all CD types, except for FECD. Patients with FECD were on average 15 ± 9 years older. The diagnosis of EBMD and FECD was based on the clinical examination (slit-lamp biomicroscopy and OCT). The diagnosis of all patients with TBCD, LCD1, GCD1, GCD2, and MCD was confirmed with genetic sequencing of TGFBI and CHST6 genes according to the methodology presented in previous author's publications [22, 23]. In the CD group eyes with differentiated severity of the disease were included in the analysis.

Clinical examination consisted of visual acuity, slit-lamp biomicroscopy with photography (magnification 10x; 16x), anterior eye segment time-domain, and spectral swept-source optical coherence tomography.

Anterior segment imaging was performed by one observer. We used two anterior segment optical coherence systems: 1310 nm time-domain OCT (TD OCT; Visante OCT; Carl Zeiss Meditec, Inc., Dublin, California, USA) and 1310 nm swept-source spectral-domain OCT (SS OCT; Casia SS-1000 OCT; Tomey, Nagoya, Japan). During the TD OCT exam, we used anterior segment (16 × 6 mm; 2 × 256 A scans), high-resolution corneal quad scans (10 × 3 mm; 4 × 512 A scans), and an automatic pachymetry map (8 × 128 A scans).

During the SS OCT exam, we used the anterior chamber angle (16 × 6 mm; 64 × 512 A scans) and cornea (10 × 4 mm; 16 × 512 A scans) protocols.

Automated and manual central corneal thickness (aCCT, mCCT), anterior chamber depth (ACD), and nasal and temporal trabecular iris angle (nTIA, tTIA) were measured. The analysis of the measurement results was performed by three observers. ACD was defined as the perpendicular distance from the corneal endothelium at the corneal apex to the anterior lens surface. TIA was defined as an angle measured with the apex in the iris recess and the arms of the angle passing through a point on the trabecular meshwork 500 *μ*m from the scleral spur and the point on the iris perpendicularly opposite [25]. In four eyes from the study group, we could not assess the scleral spur, so they were excluded from the TIA assessment. Corneal morphology assessment was performed and compared between TD OCT and SS OCT. We analyzed the characteristic features, pattern, and location of CD deposits.

Mean values and standard deviation (SD) were calculated for each parameter in the groups with more than 30 eyes (the control group, FECD). Median and range were assessed in the groups with fewer than 30 eyes (EBMD, TBCD, LCD, GCD1, GCD2, and MCD). The values for the parameters were compared between the normal and CDs groups using Student's *t*-test or the Mann-Whitney *U* test depending on the sample size. Agreement between pairs of measurements was analyzed with Bland-Altman plots. The 95% limit of agreement (mean difference ±1.96 standard deviation) was calculated. A *p* value of less than .05 was considered statistically significant.

## 3. Results 

Mean, standard deviation, and median and range values of aCCT, mCCT, ACD, nTIA, and tTIA for the control and CDs groups are presented in Table 1.

### 3.1. Agreement of aCCT, mCCT, ACD, nTIA, and tTIA Measurements

**Figure 1:**
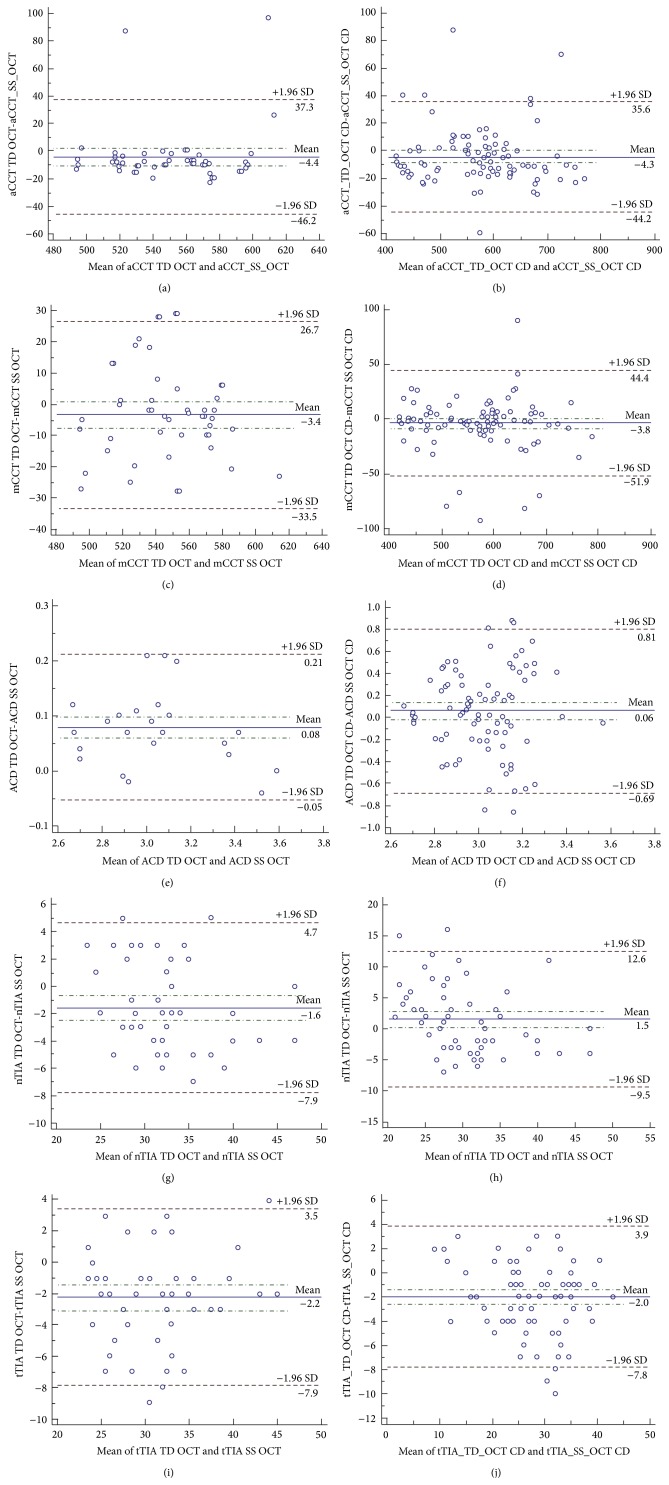
The graphic presentation of Bland-Altman plot comparing measurements of TD OCT and SS OCT in control and corneal dystrophies group (CD group). Dash-dot line: 95% CI: 95% confidence interval of the mean difference; dashed line: ±1.95 SD (standard deviation); aCCT: automated central corneal thickness; mCCT: manual central corneal thickness; ACD: anterior chamber depth; nTIA: nasal trabecular iris angle; tTIA: temporal trabecular iris angle.

Mean difference in the aCCT measurements by TD OCT and SS OCT was not statistically significant in the control group (*p* = .14) but was significant in the CD group (*p* = .04). The aCCT measured with SS OCT was on average 4.4 *μ*m higher than that measured with TD OCT in the control group and 4.32 *μ*m in the CDs group. The mCCT measurements demonstrated the best agreement between TD OCT and SS OCT with no significant difference in the control group (*p* = .12) and the CDs group (*p* = .14). The ACD measured with SS OCT was on average 0.07 mm lower than that measured with TD OCT in the control group (*p* < .001) and 0.06 mm lower in the CDs group (*p* = .01). The mean difference in the nTIA measurements was significant in both groups, *p* = .001 in the control group and *p* = .03 in the CDs group. The nTIA measured with SS OCT was 1.58° higher than that measured with TD OCT in the control group and 1.8° lower in the CDs group. The mean difference in the tTIA measurements was also significant in both groups with *p* < .001 in the control group and *p* < .001 in the CDs group. The tTIA measured with SS OCT was 2.22° higher than that measured with TD OCT in the control group and 1.97° higher in the CDs group. All data including mean difference, 95% confidence interval, standard deviation, and *p* value are presented in Table 2. The Bland-Altman plots including the 95% limit of agreement are presented in Figure 1.

### 3.2. Control Group and CDs Group Measurements Comparison

TD OCT and SS OCT measurements of aCCT and mCCT were significantly different in four corneal dystrophies (TBCD, LCD, MCD, and FECD) compared to the control group.

The aCCT and mCCT measurements were significantly higher in TBCD, LCD, and FECD compared to normal individuals. In MCD, the analysis revealed lower CCT values compared to the control group.

The mean values ± standard deviation and median values (range) of aCCT and mCCT, ACD, nTIA, and tTIA measured with TD OCT and SS OCT in the control and study group were presented in Table 1.

**Figure 2:**
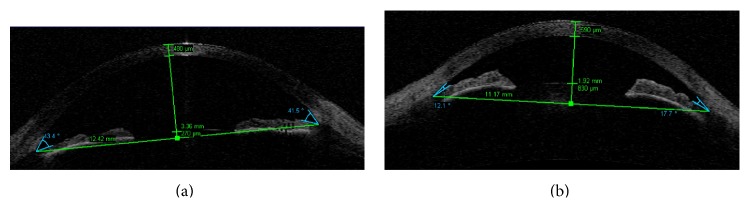
Anterior segment single 0–180° scan of TD OCT with measured results of following parameters: mCCT, ACD, nTIA, tTIA, ATA, angle to angle distance, and CLR, crystalline lens rise. (a) Control group. (b) FECD. Note the difference between mCCT, ACD, nTIA, and tTIA, which is statistically significant (*p* < .0001).

FECD was the only CD that affected all analyzed anterior eye chamber parameters (Figure 2). The aCCT, mCCT, ACD, nTIA, and tTIA measurements in FECD differed significantly from those for the control group (*p* < .001). The summary of the comparison is presented in Table 3.

### 3.3. Corneal Morphology Comparison

All corneal characteristic CD features revealed on the SS OCT scans were also visible on the TD OCT scans. That makes both techniques useful for establishing the diagnosis of each corneal dystrophy. The advantage of SS OCT is the simultaneous acquisition of numerous scans, which provides the possibility of creating a 3-dimensional corneal pattern of changes.

All corneal dystrophies deposits were hyperreflective on the TD OCT and SS OCT scans, but the level of increased reflectivity differed and extended from diffuse areas of increased reflectivity in LCD to highly reflective corneal opacities in GCD2. The opacities also differed in shape and pattern depending on the CD type.

**Figure 3:**
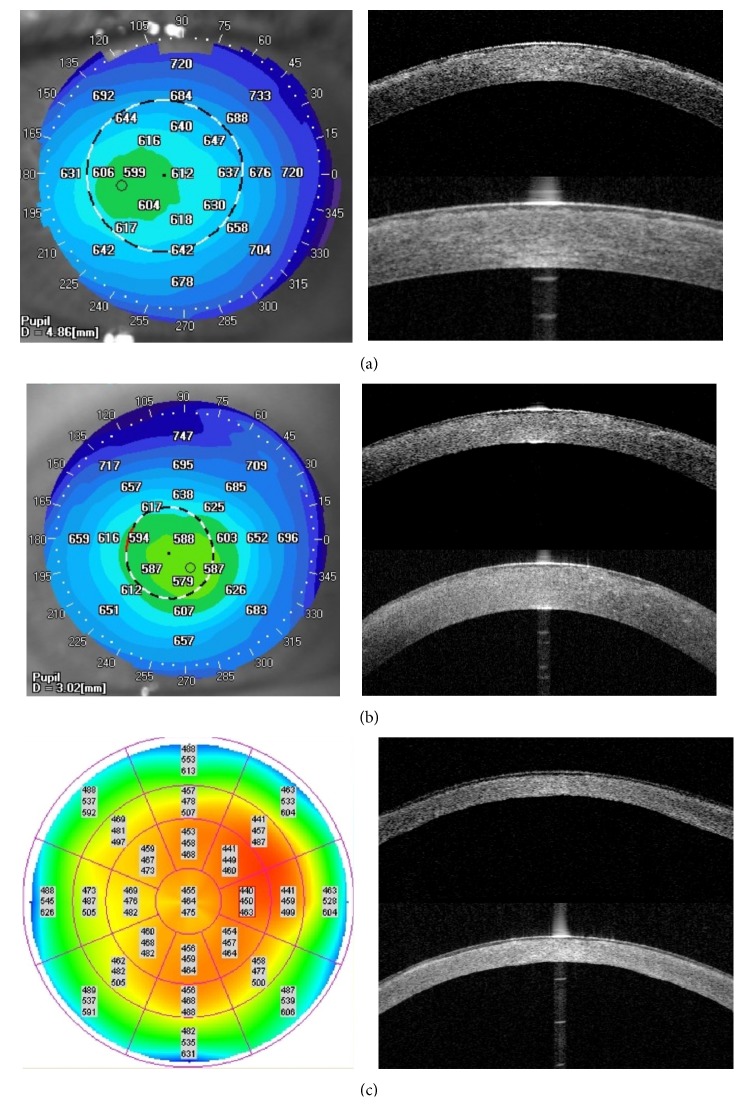
Comparison of representative TD and SS OCT corneal scans in CD group. There are no differences in corneal deposits visualization between both OCT systems. (a) TBCD: SS OCT pachymetry map showing the increase of CCT. aCCT of 612 *μ*m. TD OCT high-resolution corneal scan and SS OCT cornea scan showing increased reflectivity in the area of Bowman layer and anterior corneal stroma. The deposits are causing the irregularity of the anterior stromal border from the epithelium side. (b) LCD: SS OCT pachymetry map showing the increase of CCT. aCCT of 588 *μ*m. TD OCT high-resolution corneal scan and SS OCT cornea scan presenting diffuse areas of increased reflectivity in the area of Bowman layer and anterior to mid stroma. (c) MCD: TD OCT pachymetry map indicating corneal thinning with aCCT of 464 *μ*m. TD OCT high-resolution corneal scan and SS OCT cornea scan showing general increased reflectivity throughout the corneal stroma. Note the irregularity of the anterior stromal border from the epithelium side and the diffuse areas of hyperreflectivity in Bowman's layer. There is a noticeable flat layer of increased reflectivity in the posterior, peripheral corneal part.

No changes in EBMD were distinguishable on either OCT scan. TBCD was characterized by increased reflectivity in the Bowman layer and anterior corneal stroma (Figure 3). The deposits caused the irregularity of the anterior stromal border from the epithelium side forming a sawtooth pattern of hyperreflective material. LCD caused diffuse areas of increased reflectivity in the area of Bowman layer and anterior to midstroma. GCD1 was characterized by focal granular hyperreflective changes in the Bowman layer and anterior to mid corneal stroma. Corneal deposits in GCD2 had the highest reflectivity; there were highly reflective, flat corneal opacities in the anterior stroma accompanied by focal deposits located in the midstroma. MCD caused general increased reflectivity throughout the corneal stroma. The deposits caused the irregularity of the anterior stromal border from the epithelium side and the diffuse areas of hyperreflectivity in Bowman's layer. There was a noticeable flat layer of increased reflectivity in the posterior, peripheral corneal part. FECD caused corneal edema that was characterized by irregularity of the posterior corneal border and corneal epithelial and subepithelial bullae in advanced stages.

## 4. Discussion

According to the authors of the IC3D classification system (the International Committee for Classification of Corneal Dystrophies), understanding of corneal dystrophies is still evolving due to the development of noninvasive imaging techniques and introduction of genetic testing [26]. OCT provides direct, noncontact, anterior eye segment imaging allowing morphology and morphometry analysis. SS OCT scans 360° around the anterior segment in 2.4 sec showed the depth and extent of the pathologic corneal features. Good repeatability and reproducibility of SS OCT anterior eye segment measurements were proved in normal controls [27–31]. Pachymetric maps made with SS OCT were compared with a rotating Scheimpflug camera, ultrasound pachymetry, specular microscopy, slit-scanning topography, TD OCT, and 830 nm SD OCT with high correlation rates [13, 17, 22–27]. Fukuda et al. revealed that the CCT measured with Scheimpflug camera was significantly larger than that measured with SS OCT, slit-scanning topography, and ultrasonic pachymetry (*p* < .001) [28]. Fukuda et al. revealed that CCT measured with SS OCT was thinner compared with slit-scanning topography (*p* < .001) and ultrasound pachymetry (*p* < .001) [29]. The authors emphasize that CCT values measured with different devices are not interchangeable. Anterior chamber angle parameters such as TIA, TISA 500, 750 (trabecular iris space area at 500, 750 *μ*m from the scleral spur) and AOD 500, 750 (angle opening distance at 500, 750 *μ*m from the scleral spur) measurements repeatability, reproducibility, and agreement between SS OCT and other devices were studied in normal and glaucomatous eyes, but no such studies have been conducted for opaque corneas [14–16, 30, 31]. SS OCT demonstrated the high reproducibility of angle analysis in healthy subjects.

Our study confirms the good agreement of CCT measurements between devices in healthy subjects (aCCT, *p* = .4; mCCT, *p* = .12). The ACD, nTIA, and tTIA measurements differed significantly. ACD values measured with SS OCT were significantly lower (mean difference = 0.07 ± 0.06 mm; *p* < .001). nTIA and tTIA measured with SS OCT were significantly larger (mean difference = 1.58 ± 3.2°; *p* = .001; mean difference = 2.22 ± 2.91°; *p* < .001, resp.). Fukuda et al., who studied agreement of CCT, ACD, and anterior chamber width measurements in 85 normal individuals between the TD OCT and SS OCT prototype, also revealed no statistically significant difference in CCT measurements. ACD measurements were significantly different (*p* < .001); the mean difference was 0.04 mm smaller compared to our study [28]. Aptel et al. studied CCT, ACD, TIA, TISA 500, 750, and AOD 500, 750 measurements in healthy subjects. The study revealed that ACD measured with SS OCT was significantly larger (mean difference = 0.12 ± 0.08 mm; *p* < .001), and the TIA measured with the SS OCT was significantly lower (mean difference = 4.85° ± 5.30°; *p* < .01). There were nonsignificant differences between the devices for the other parameters (*p* > .06) [30]. The opposite results of the ACD and TIA measurements found by Aptel et al. and our study indicate that there is no systematic difference in ACD and TIA measurements between TD and SS OCT, and the results could vary depending on the device used for measurements.

To date, no comparison of CCT, ACD, and TIA measurements with TD OCT and SS OCT has been published for opaque corneas. Accurate pachymetric and angle measurements in an eye with a corneal opacity are challenging and of great importance in guiding treatment or retreatment in corneal surgeries. Overestimation or underestimation in anterior segment parameters could be misleading in selecting the corneal transplant type as well as deciding the depth of treatment in phototherapeutic keratectomy. The 830 and 1310 nm OCT was proved useful in the selection and planning of surgical procedures to treat GCD by determining the size, depth, and location of deposits [24, 32]. We revealed that only the mCCT showed good agreement between TD OCT and SS OCT in the CDs group (*p* = .14). There were significant differences for other studied parameters. That result should be considered in clinical practice, while planning surgical treatment in CD. It emphasizes the role of corneal manual measurements in establishing the treatment plan.

Our study also indicated the impact of CD on corneal pachymetry and anterior chamber parameters. TBCD, LCD, and FECD caused increased aCCT and mCCT measured with both devices compared to the control group. MCD was characterized by significant corneal thinning, indicated by previous studies [23, 33]. The increase in CCT is the main feature of moderate and advanced FECD, but it is possible that association with other anterior eye segment parameters change is rarely examined. A link of FECD to axial hypermetropia, shallow anterior chamber, and angle closure glaucoma was suggested in the 1990s [34, 35], but another study found no significant difference in ACD between patients and controls [36] and it was not further confirmed with OCT studies. Our study indicated a significant increase in CCT, thus indicating the advanced stage of FECD and the significant decrease of ACD, nTIA, and tTIA in all 31 patients. The significant ACD and TIA change probably is the result of the increase in CCT, which was proved to be one factor associated with narrow ACD and angle closure glaucoma in the Beijing Eye Study 2006 [37].

Regarding CD corneal morphology analysis, our current SS OCT study complements previous findings demonstrated based on TD, SD, and SS OCT [19–23, 32, 33].

The weak part of our study was that including different stages of the CD could affect the outcomes. On the other side, due to the rarity and the individual course of the disease among patients, further division of the study group into subgroups would result in the insufficient number of subjects for statistical analysis.

To conclude, better agreement between SS OCT and TD OCT measurements was demonstrated in normal individuals compared to the CDs group. Our study emphasizes the role of manual measurements in establishing corneal thickness in CDs. OCT provides comprehensive corneal deposits analysis and demonstrates the association of CD with CCT, ACD, and TIA measurements.

## Figures and Tables

**Table 1:** Results of automated and manual central corneal thickness (aCCT, mCCT), anterior chamber depth (ACD), and nasal and temporal trabecular iris angle (nTIA, tTIA) measurements by swept-source optical coherence tomography SS OCT and time-domain optical coherence tomography TD OCT. Values were calculated as mean ± standard deviation (SD) or median and range depending on the sample size (<30 or ≥30). BCVA results were presented as range for all groups. EBMD = epithelial basement membrane dystrophy, TBCD = Thiel-Behnke corneal dystrophy, LCD1 = lattice corneal dystrophy TGFBI type, GCD1 = granular corneal dystrophy type 1, GCD2 = granular corneal dystrophy type 2, MCD = macular corneal dystrophy, and FECD = Fuchs endothelial corneal dystrophy.

Parameter	OCT device	Control group	Study group						

CD type			EBMD	TBCD	LCD	GCD1	GCD2	MCD	FECD
Number of eyes		50 eyes	12 eyes	6 eyes	15 eyes	7 eyes	2 eyes	23 eyes	31 eyes

BCVA		1.0	1.0	0.3–0.9	0.1–0.5	0.05–0.8	0.5–0.6	0.05–0.2	0.05–0.4

aCCT [*µ*m]	TD OCT	548.96	545	600	583	550	538.5	459	675.54
		±37.34	524–586	587–615	550–620	498–567	529–548	414–492	±42.19
	SS OCT	553.96	554.5	598.5	587	546	528	453	682.03
		±31.91	518–600	578–620	546–619	480–583	518–538	419–502	±41.38

mCCT [*µ*m]	TD OCT	546.94	541.5	602	588	555	530	456	676.03
		±31.13	517–582	587–618	528–610	490–578	520–540	417–503	±43.13
	SS OCT	550.34	553.5	599.5	593	550	513	447	675.45
		±31.13	507–580	580–625	550–621	498–576	507–519	418–508	±52.98

ACD [mm]	TD OCT	3.0514	3.085	2.9	3.13	3.11	3.22	3.05	2.30
		±0.24	2.71–3.45	2.71–3.15	2.71–3.59	2.72–3.24	3.06–3.39	2.61–3.59	±0.35
	SS OCT	2.9718	2.955	2.91	3.015	2.98	3.18	2.98	2.36
		±0.25	2.61–3.38	2.61–3.05	2.61–3.59	2.68–3.05	3.01–3.36	2.69–3.59	±0.46

nTIA [°]	TD OCT	31.44	31	27	30	33	33	31	21.38
		±4.98	24–39	24–32	24–47	29–38	30–36	24–41	±4.40
	SS OCT	33.02	33.5	26.5	33	34	30.5	32	19.61
		±5.67	22–42	22–39	26–47	32–42	27–34	20–42	±4.49

tTIA [°]	TD OCT	29.9	27.5	27	29	32	30	30	19.38
		±5.81	22–42	23–34	22–39	29–41	26–34	24–37	±5.33
	SS OCT	32.12	31.5	30	33	34	32.5	31	20.29
		±5.57	24–44	24–36	23–40	30–40	29–36	24–41	±6.01

**Table 2:** Bland-Altman plot comparing automated and manual central corneal thickness (aCCT, mCCT), anterior chamber depth (ACD), and nasal and temporal trabecular iris angle (nTIA, tTIA) measurements by swept-source optical coherence tomography SS OCT and time-domain optical coherence tomography TD OCT in control and corneal dystrophies group (CD group). 95% CI:95% confidence interval of the mean difference; SD: standard deviation.

	Control group				CD group			
	Mean difference	95% CI	SD	*p*	Mean difference	95% CI	SD	*p*
aCCT	−4.4	−10.47–1.63	21.29	.14	−4.32	−8.51–0.13	20.35	**.04**
mCCT	−3.4	−7.7–0.97	15.37	.12	−3.75	−8.81–1.31	24.58	.14
ACD	0.07	0.06–0.09	0.06	<**.001**	0.06	−0.01–0.14	0.38	**.01**
nTIA	−1.58	−2.48–−0.67	3.2	**.001**	1.8	0.14–3.47	6.62	**.03**
tTIA	−2.22	−3.04–−0.39	2.91	<**.001**	−1.97	−2.59–−1.36	2.97	<**.001**

**Table 3:** Statistical difference of anterior eye segment measurements: automated and manual central corneal thickness (aCCT, mCCT), anterior chamber depth (ACD), and nasal and temporal trabecular iris angle (nTIA, tTIA) measurements by swept-source optical coherence tomography SS OCT and time-domain optical coherence tomography TD OCT comparing different corneal dystrophies and normal eyes. EBMD = epithelial basement membrane dystrophy, TBCD = Thiel-Behnke corneal dystrophy, LCD1 = lattice corneal dystrophy TGFBI type, GCD1 = granular corneal dystrophy type 1, GCD2 = granular corneal dystrophy type 2, MCD = macular corneal dystrophy, and FECD = Fuchs endothelial corneal dystrophy.

Statistical difference	Study group versus normal controls					
	EBMD	TBCD	LCD	GCD1	MCD	FECD
aCCT						
TD OCT	*p* .89	**p** < .001	**p** < .001	*p* .82	**p** < .001	**p** < .001
	*U* 292.5	*U* 17.5	*U* 144.5	*U* 166	*U* 6	
SS OCT	*p* .93	**p** < .001	**p** < .001	*p* .24	**p** < .001	**p** < .001
	*U* 295.5	*U* 48.41	*U* 170.5	*U* 127.5	*U* 13	
mCCT						
TD OCT	*p* .72	**p** < .001	**p** < .001	*p* .85	**p** < .001	**p** < .001
	*U* 281.5	*U* 8	*U* 128	*U* 167.5	*U* 9.5	
SS OCT	*p* .77	**p** < .001	**p** < .001	*p* .84	**p** < .001	**p** < .001
	*U* 284	*U* 20	*U* 94	*U* 167	*U* 4.5	
ACD						
TD OCT	*p* .83	*p* .24	*p* .33	*p* .55	*p* .87	**p** < .001
	*U* 288.5	*U* 106.5	*U* 335	*U* 150.5	*U* 562	
SS OCT	*p* .83	*p* .53	*p* .30	*p* .75	*p* .53	**p** < .001
	*U* 288.5	*U* 126.5	*U* 324.5	*U* 159	*U* 51	
nTIA						
TD OCT	*p* .93	*p* .06	*p* .69	*p* .25	*p* .98	**p** < .001
	*U* 295.5	*U* 81	*U* 374	*U* 128.5	*U* 573.5	
SS OCT	*p* .84	*p* .07	*p* .70	*p* .12	*p* .49	**p** < .001
	*U* 289	*U* 83.5	*U* 375	*U* 112	*U* 517	
tTIA						
TD OCT	*p* .68	*p* .27	*p* .65	*p* .11	*p* .72	**p** < .001
	*U* 277	*U* 109	*U* 370	*U* 110	*U* 545.5	
SS OCT	*p* .95	*p* .30	*p* .91	*p* .23	*p* .93	**p** < .001
	*U* 297	*U* 111.5	*U* 393	*U* 126	*U* 568.5	
